# Cultural capital, the digital divide, and the health of older adults: a moderated mediation effect test

**DOI:** 10.1186/s12889-024-17831-4

**Published:** 2024-01-25

**Authors:** Yupeng Cui, Youshi He, Xinglong Xu, Lulin Zhou, Jonathan Aseye Nutakor, Lingqing Zhao

**Affiliations:** School of Management, Jiangsu University, Zhenjiang, Jiangsu Province China

**Keywords:** Cultural capital, Digital divide, Cognitive ability, Health of older adults

## Abstract

**Background:**

It is of great practical significance to study the intrinsic relationship between cultural capital, digital divide, cognitive ability, and health of older adults in the dual social context of population aging and the digital era.

**Methods:**

We analyzed data from the 2020 China Family Panel Studies (CFPS) initiated by the China Center for Social Science Surveys at Peking University. Physical health, mental health, and memory health were set as indicators of older adults, and the relationship between cultural capital, digital divide, cognitive ability, and health of older adults was examined by hierarchical regression with moderated mediated effect methods.

**Results:**

Improvement in the health of older adults is associated with an increase in the level of cultural capital; cultural capital may bridge the digital divide faced by older adults, which in turn promotes the improvement of the health of older adults; the higher the level of cognitive ability, the stronger the effect of cultural capital on the digital divide, and at the same time, the stronger the mediating effect of the digital divide; cultural capital has a more pronounced effect on the health of older male adults living in the city.

**Conclusions:**

The results of the study show that cultural capital can have a positive impact on the health of older adults, but there is urban-rural heterogeneity and gender heterogeneity, in which the digital divide plays a mediating role, and the enhancement of the cognitive ability of older adults will be conducive to the improvement of their health, so the health of older adults should be promoted by improving the level of their cultural capital and the ability of older adults to use digital technology, thus provide references for the protection of health of older adults.

## Background

Health is the basis for individuals to participate in social activities and the source of their life activities, and health has always been a primary concern. According to data released by the National Bureau of Statistics, by the end of 2022, the number of older adults aged 60 and above in China will have exceeded 280 million, and the proportion of older adults aged 65 and above will be 14.9%, further deepening the degree of aging. At the same time, the health of older adults is not optimistic; according to the latest data released by the National Health and Health Commission, the proportion of older adults suffering from at least one chronic disease is as high as 78% [1]. With the increase of age, the health of older adults in terms of sensory, cognitive, physical, and psychological aspects is becoming increasingly severe, and the health of older adults has become the focus of people’s attention. In order to cope with the problem of population aging, the government issued the National Medium- and Long-Term Plan for Actively Responding to Population Aging, which takes actively responding to population aging as a national strategy and encourages the integration and participation of older adults in social activities through a variety of practical means, better to protect the level of health of older adults [2]. Having a good health condition in older adults not only has a positive impact on their peaceful enjoyment of their old age but also has an essential impact on easing the pressure of social old age. Therefore, solving the health of older adults is a requirement for guaranteeing the quality of life of older adults and a task of the times to cope with the aging society actively.

Health is a multidimensional concept, and many factors affect the health of older adults. Some scholars explore the influence of behavioral habits (such as physical exercise, etc.) on the health of older adults at the current stage. They believe good behavioral habits may help older adults improve their health and sleep quality [3]. Some scholars also believe that childhood migration experience will have an impact on the health of individuals, and childhood migration experience will affect the cognitive ability of individuals, and individuals with childhood migration experience are more likely to have mental health problems than those without migration experience in the same year and the likelihood of suicide will be significantly increased [4]. Some scholars also take the perspective of social environment and believe that the living environment and working conditions in which individuals live also impact their health, which is the leading cause of health inequality [5]. In addition to this, scholars have also studied the influencing factors on the health of older adults from the aspects of social support, social capital, welfare level, and health insurance [6–9]. It can be seen that cultural capital has been less studied in the influencing factors of the health of older adults, and the unequal distribution of cultural capital can bring about problems such as the unequal distribution of social resources, which in turn adversely affects individual health [10]. Billings et al. used a homemade questionnaire to investigate the students of a university, and the study found that family cultural capital has a significant positive effect on the mental health of college students [11]. Cultural capital is an essential mechanism for coping with social inequality and attenuating social complexity.

Cultural capital was first proposed by French sociologist Bourdieu, who believed that there are three types of cultural capital: one exists in the subjective form of cultural cultivation, temperament, etc.; one exists in the solid form of books, implements, etc.; and one exists in the institutionalized form of academic qualifications, certificates, etc. [12].. Cultural capital and social capital are interconnected and influence each other. Cultural capital is a prerequisite for individuals to accumulate social capital, and individuals with sufficient cultural capital can better carry out social interactions and social activities. The theory of cultural capital has been proven to be an effective tool for criticizing the problem of unequal distribution of social resources and social discrimination [13]. Later, scholars enriched and developed the connotation of cultural capital proposed by Bourdieu and further divided cultural capital into individualized, objectified, and institutionalized cultural capital [14].

Data from *the 50th Statistical Report on the Development of the Internet in China* shows that as of June 2022, China’s Internet penetration rate reached 74.4%, and the number of Internet users reached 1.051 billion, but the number of elderly Internet users aged 60 and above accounted for only 11.3%. In the age structure of China’s population, older adults aged 65 and above already account for more than 14%. The number of elderly Internet users is obviously lower than the proportion of its population. As a “digitally disadvantaged group,” older adults lack digital literacy, information literacy, knowledge literacy, and so on, which is contrary to the requirements of the development of the Internet era, and the problem of the digital divide among older groups is more prominent. The emergence of the digital divide problem is related to older adults’ weak access to and mastery of information, which has become the primary basis for social stratification in the Internet era [15]. The so-called digital divide mainly refers to the difference between those who use the Internet and those who do not [16]. Based on the basic concept, scholars divided the digital divide into three parts: the first digital divide (access gap), the second digital divide (usage gap), and the third digital divide (knowledge gap). Meanwhile, the degree of harm of these three digital divides was studied, and it was found that the usage gap is more harmful and more likely to negatively impact society [17]. Some studies have shown that despite the rapid development of mHealth, which allows older adults to access resources about improving their health, the digital divide prevents older adults from utilizing mHealth to access information about their health, which ultimately has a negative impact on their health [18]. Research by scholars such as Leguina has shown that cultural capital significantly affects the solution of the digital divide problem [19]. The cultural capital of older adults can impact the digital divide problem through continuous accumulation and transformation. In the Internet era, older adults’ cognitive ability of Internet technology is biased, and their acceptance of new things is not strong. The cognitive ability of older adults will not only impact the accumulation of cultural capital but also impact the problem of the digital divide and the physical and mental health faced by older adults. Therefore, this paper incorporates the digital divide and cognitive ability into the analytical framework.

Stress coping theory emphasizes that individuals take measures (positive or negative) to resolve the conflict between stressors and individuals. Older adults can also positively impact their physical and mental health by increasing their stock of cultural capital and other positive measures to cope with the problem of the digital divide they face in the Internet age [20]. This leads to this paper’s reflection: What is the effect of cultural capital on the health of older adults? How is the heterogeneity? What are the mechanisms of the digital divide and cognitive ability? Based on this, this paper uses data from the China Family Panel Studies (CFPS) to explore the influence effect of cultural capital on the health of older adults. Further, it analyzes the mediating effect of the digital divide and the moderating effect of cognitive ability, to provide references for the improvement of the health of older adults. The possible marginal contributions of this paper are, first, to analyze the pathways to protect the health of older adults from the perspective of their cultural capital, and to enrich the antecedent research on the health of older adults; second, incorporating the digital divide and cognitive ability into the analytical framework, adopting hierarchical regression and moderated mediated effect test methods to empirically explore the impact of cultural capital on the health of older adults, and analyzing the roles played by the digital divide and cognitive ability in it, enriching the related research on the health of older adults; third, under the dual social background of population aging and the digital era, the positive effects of cultural capital on the health of older adults are revealed using a study sample of 6,619 older adults in China, providing empirical evidence for the protection of the health of older adults, the active response to population aging, and the strategy of building a healthy China.

## Methods

### Data sources

The China Family Panel Studies (CFPS) database is conducted by the China Social Science Survey Center of Peking University, using a multi-stage, implicitly stratified, systematic probability sampling method proportional to the population size, with the questionnaire divided into four parts: a questionnaire for children, a questionnaire for villagers, a questionnaire for adults, and a questionnaire for households. Taking the 2010 survey data as the baseline, the questionnaire is updated every two years, covering a wide range of areas such as household income, education, employment, Internet use, and health, etc., in China, which provides essential data support for public policy and population research. In this paper, data from the Chinese Family Panel Study (CFPS) were selected according to the needs of the research objectives, and the data of older adults under the age of 60 were excluded, and after deleting the samples of older adults containing missing variables and invalid variables, a total of 6619 valid samples were obtained.

### Variable description

#### Outcome variables

Health is characterized by multidimensionality, so health measurement indicators are diverse. Health is divided into three dimensions: physical health, mental health, and memory health [21], and health should refer to the overall health status of an individual, including physical, mental, and memory health. Self-assessed health refers to an individual’s assessment of his or her condition, which is a good reflection of the individual’s physical health [22]. Measured by the question “How do you think your health is?“, the options are divided into “very healthy, quite healthy, relatively healthy, general, unhealthy,” and the reverse assignment method is used to assign the value (1–5 points), the higher the score, the better the health condition. The higher the score, the better the health condition. Mental health is defined as the psychological well-being of an individual in carrying out social activities. The question in the questionnaire “In the past week, I have felt depressed” was used as the measurement criterion, and the options were categorized as “hardly ever (less than a day), some of the time (1–2 days), often (3–4 days), and most of the time (5–7 days),” and the higher the score, the better the psychological well-being. The exact reverse assignment method was used to assign values (1–4 points), with higher scores indicating better mental health. Memory health refers to the extent to which an individual remembers things that happen, as measured by the question in the questionnaire, “How many of the main things that happened to you in a week can you remember?” with the option “Only a little bit, only a few, half, most, and completely,” each assigned a score of 1–5, with higher scores indicating better memory health.

#### Independent variables

The independent variable in this paper is cultural capital. Since less objective cultural capital is involved in the China Family Panel Studies (CFPS) data, which can lead to problems of retention, this paper analyzes both individual and institutional cultural capital dimensions. Institutional cultural capital is mainly reflected through the education received by the individual. The education level can be a good reflection of the specific content, measured by the questionnaire title “The highest level of education has been completed,” the title will be transformed into “Have you received an education,” “No” corresponds to “illiterate/semi-literate, never attended school,” “Yes” corresponds to “primary school, junior high school, junior high school, technical school, vocational high school, college, bachelor’s degree, master’s degree, doctoral degree.” “Yes” is scored as 1 point, and “No” is scored as 0 points. Individual cultural capital is mainly expressed in individuals’ characteristics and behavioral habits, which are reflected through participation in cultural activities. The question “Have you read any books in the past 12 months?” in the questionnaire was used as a measure, and a “yes” answer was assigned a score of 1, while a “no” answer was assigned a score of 0. The higher the score, the higher the individual’s cultural capital level.

#### Mediating variables

Digital divide is the mediating variable in this paper. Drawing on Ran’s approach [23], “whether to use a computer to access the Internet” and “whether to use a mobile device to access the Internet” in the questionnaire were used as indicators of the digital divide. If the answer is “yes”, it will be assigned a value of 1, and vice versa, it will be assigned a value of 0. The total score ranges from 0 to 2, and the higher the score, the better the ability to use the Internet and the better the effect of bridging the digital divide.

#### Moderating variables

The moderating variable in this paper is the cognitive ability of older adults. Cognitive ability is the ability of an individual to adapt to his or her environment, learn from experience, understand complex ideas and apply what he or she has learned to overcome difficulties [24]. However, in conjunction with the purpose and research context of this study, cognitive ability is defined in this study as the ability of older adults to assess the importance of obtaining information resources on the Internet, which can reflect the degree of adaptation of older adults to the digital society, as well as their motivation to make efforts to learn about the Internet in order to adapt to the digital society. Only when older adults realize the importance of the Internet for obtaining information resources, the higher their level of cognitive ability and motivation to learn and use the Internet. The questionnaire “The Importance of the Internet in obtaining information” is used as the basis for measuring the cognitive ability of older adults. The higher the score, the more importance older adults attach to the Internet, the better their cognitive ability, and the more motivated they are to learn and use the Internet.

#### Control variables

In order to reduce the omitted variable bias, the following control variables are selected in this paper: essential personal characteristics, selected variables such as household, gender, age, and marriage status; medical insurance factors, selected variables such as participation in health insurance status; life habit factors, selected variables such as smoking and alcohol abuse; and family economic factors, selected variables such as annual income, pension, and children’s giving. The variable assignments and descriptive statistics are shown in Table 1.

**Table 1:** Variable assignments and descriptive statistics

Variables		Mean value	Standard deviation	Minimum value	Maximum value
Health of older adults	Physical health	2.650	1.261	1	5
	Mental health	3.279	0.859	1	4
	Memory health	2.576	1.335	1	5
Cultural capital	Individual cultural capital	0.130	0.126	0	1
	Institutional cultural capital	0.615	0.487	0	1
Digital divide	Mobile device access	0.239	0.427	0	1
	Internet access	0.030	0.170	0	1
Cognitive ability	Importance of the Internet	2.178	1.516	1	5
Control variables	Household	0.498	0.500	0	1
	Gender	0.482	0.500	0	1
	Age	1.311	0.539	1	4
	Marriage	0.867	0.340	0	1
	Income	2.672	1.202	0	5.602
	Health insurance	0.905	0.294	0	1
	Smoke	0.281	0.450	0	1
	Drink	0.161	0.367	0	1

#### Model setting

This paper draws on the analytic process with moderated mediation proposed by Hayes (2018) to sequentially test the direct effect of cultural capital on the health of older adults, the mediating effect of the digital divide, the moderating effect of cognitive ability, and the moderated mediating effect. The specific test is divided into four steps:

1 $$ Y={c}_{0}+{c}_{1}W+{c}_{2}R+{c}_{3}WR+{\mu }_{1}$$

2 $$ D={a}_{0}+{a}_{1}W+{a}_{2}R+{a}_{3}WR+{\mu }_{2}$$

3 $$ Y={c}_{0}^{{\prime }}+{c}_{1}^{{\prime }}W+{c}_{2}^{{\prime }}R+{c}_{3}^{{\prime }}WR+{b}_{1}D+{b}_{2}RD+{\mu }_{3}$$

In Eqs. (1), (2), and (3), $$ Y$$ denotes the health of older adults; $$ W$$ denotes cultural capital; $$ R$$ denotes cognitive ability; $$ D$$ denotes digital divide; and $$ {\mu }_{1}$$, $$ {\mu }_{2}$$, and$$ {\mu }_{3}$$ are error terms. The first step tests the direct effect, i.e., to determine whether the coefficient of $$ {c}_{1}$$ in Eq. (1) is significant or not, and $$ {c}_{1}$$ is the direct effect without considering the mediator (the direct effect of cultural capital on the health of older adults). The second step tests the mediating effect. The coefficient $$ {a}_{1}$$ in Eq. (2) is the first half of the mediating effect (the effect of cultural capital on the digital divide), and the coefficient $$ {b}_{1}$$ in Eq. (3) is the second half of the mediating effect (the effect of the digital divide on the health of older adults). The mediating effect requires determining whether the coefficients of the front and back segments are individually significant and verifying the significance of the product of coefficients $$ {a}_{1}{b}_{1}$$. The third step tests for moderating effects, where $$ {c}_{3}$$ is the coefficient of the moderating effect on the direct effect path (i.e., the moderating effect of cognitive ability on the direct effect of cultural capital and health of older adults), $$ {a}_{3}$$ is the coefficient of the moderating effect on the first half of the mediating path (i.e., the moderating effect of cognitive ability between cultural capital and the digital divide), and $$ {b}_{2}$$ is the coefficient of the moderating effect on the second half of the mediating path (i.e., the moderating effect of cognitive ability between the digital divide and health of older adults). The fourth step tests for a moderated mediating effect, i.e., whether the mediating effect of the digital divide is moderated by cognitive ability. Substituting (2) into (3), the moderated mediation effect is $$ {a}_{1}{b}_{1}+{a}_{1}{b}_{2}R+{a}_{3}{b}_{1}R+{a}_{3}{b}_{2}{R}^{2}$$, which can be split into two parts: the mediation effect $$ {a}_{1}{b}_{1}$$ and the moderating variable $$ R$$-related to the terms $$ {a}_{1}{b}_{2}$$, $$ {a}_{3}{b}_{1}$$, $$ {a}_{3}{b}_{2}$$, and the coefficients of any of these three groups can be verified by the test of significance that the moderated mediation effect exists.

## Results

This paper analyzed the data using hierarchical regression, and all regressions used heteroskedasticity robust standard errors. Multicollinearity diagnosis was first performed on all regression models. The purpose of the multicollinearity diagnosis is to test whether there is a high degree of correlation between the variables, and if the correlation between the variables is high it will affect the results of the study. For example, sociologists usually correlate education, income, wealth and social status measures to measures socio-economic status. In this study, education and income are jointly included in the regression model, which needs to be tested for multicollinearity so as to ensure the scientific validity of the results of this paper. The analysis results showed that the variance inflation factor (VIF) of each model was well below 3.0, and the tolerance values were all greater than 0.1, indicating that the data did not suffer from the problem of multicollinearity and that the study could be continued.

### Direct effects of cultural capital on the health of older adults

Table 2 Eq. 1 and Eq. 2 show the results of the test of the direct effect of cultural capital on the health of older adults, only the control variable is added to Eq. 1, and the independent variable cultural capital is added to Eq. 2 on the basis of Eq. 1, and Eq. 2 shows that cultural capital has a significant positive effect on the health of older adults (*β* = 1.153, *P* < 0.01). In addition, by observing the amount of change in R^2^ between Eq. 1 and Eq. 2, it can be found that Eq. 2 has an increase of 0.023 in R^2^ compared to Eq. 1, which indicates that cultural capital has a strong explanatory power on the health of older adults.

**Table 2:** Hierarchical regression test results

Variables	Dependent variable: Health of older adults					Dependent variable: Digital divide		
	Equation 1	Equation 2	Equation 3	Equation 4	Equation 5	Equation 6	Equation 7	Equation 8
Cultural capital		1.153^***^ (0.090)	0.975^***^ (0.154)	0.907^***^ (0.094)	0.908^***^ (0.094)	0.231^***^ (0.009)	0.163^***^ (0.008)	0.001 (0.014)
Cognitive ability			0.089^***^ (0.029)	0.044^**^ (0.021)	0.038 (0.023)		0.067^***^ (0.002)	0.037^***^ (0.003)
Cultural capital × cognitive ability			0.033 (0.055)					0.072^***^ (0.005)
Digital divide				0.875^***^ (0.132)	0.712^**^ (0.302)			
Digital divide × cognitive ability					0.049 (0.082)			
Household	0.573^***^ (0.056)	0.430^***^ (0.057)	0.415^***^ (0.056)	0.349^***^ (0.057)	0.349^***^ (0.057)	0.086^***^ (0.006)	0.076^***^ (0.005)	0.075^***^ (0.005)
Gender	-0.618^***^ (0.067)	-0.387^***^ (0.068)	-0.381^***^ (0.068)	-0.390^***^ (0.068)	-0.389^***^ (0.068)	0.007 (0.007)	0.013^**^ (0.006)	0.008 (0.006)
Marriage	0.118 (0.083)	0.093 (0.082)	0.088 (0.082)	0.084 (0.082)	0.085 (0.082)	0.007 (0.008)	0.004 (0.008)	0.005 (0.007)
Age	-0.058 (0.052)	-0.025 (0.052)	0.014 (0.052)	0.038 (0.052)	0.037 (0.052)	-0.053^***^ (0.005)	-0.027^***^ (0.005)	-0.026^***^ (0.005)
Income	0.177^***^ (0.023)	0.135^***^ (0.023)	0.129^***^ (0.023)	0.117^***^ (0.023)	0.117^***^ (0.023)	0.018^***^ (0.002)	0.015^***^ (0.002)	0.014^***^ (0.002)
Health insurance	0.206^**^ (0.094)	0.156^*^ (0.092)	0.152^*^ (0.092)	0.135 (0.092)	0.137 (0.092)	0.022^**^ (0.009)	0.019^**^ (0.009)	0.020^**^ (0.008)
Smoke	0.075 (0.082)	0.124^*^ (0.071)	0.130^*^ (0.071)	0.124^*^ (0.071)	0.124^*^ (0.071)	0.004 (0.007)	0.008 (0.007)	0.007 (0.006)
Drink	0.330^***^ (0.080)	0.349^***^ (0.079)	0.360^***^ (0.078)	0.369^***^ (0.078)	0.368^***^ (0.078)	-0.017^**^ (0.008)	-0.009 (0.007)	-0.011 (0.007)
R^2^	0.061	0.084	0.089	0.094	0.094	0.184	0.335	0.356
Adjusted R^2^	0.060	0.083	0.087	0.093	0.093	0.183	0.334	0.355
F	54.077^***^	67.594^***^	58.324^***^	62.640^***^	57.444^***^	165.211^***^	333.420^***^	331.478^***^

*Note*: ^***^, ^**^, and ^*^ indicate 1%, 5%, and 10% significance levels, respectively; heteroskedasticity robust standard errors are in parentheses; the health of older adults is calculated as the arithmetic mean of physical health, mental health and memory health (same below)

### The mediating effects of the digital divide

The test results of the mediating effect of the digital divide are presented in Eq. 4 and Eq. 6 in Table 2. Equation 6 is the first half of the test of the mediating effect. The results show that the higher the level of cultural capital, the smaller the problem of digital divide faced by the elderly (*β* = 0.231, *P* < 0.01). Equation 4 shows the second half of the test for mediating effects. The results showed that the smaller the digital divide problem faced by older adults, the higher their health of older adults (*β* = 0.875, *P* < 0.01). This paper uses Bootstrap to further test the mediating effect of digital divide to address the low testing power of the stepwise regression test coefficient method. Table 3 shows the test results of Bootstrap, from which it can be seen that the confidence interval of the indirect effect of the digital divide at the 95% level is [0.175, 0.288], which does not contain 0, indicating that the digital divide plays a part of the mediating effect in the process of cultural capital affecting the health of older adults. The results of estimating the intervals of the various dimensions of cultural capital show that the mediating effect of the digital divide can still pass the significance test, and the confidence interval does not contain 0. In addition, this study tested the mediating effect of the digital divide between cultural capital (and the dimensions of cultural capital) and health of older adults in urban and rural areas, and the results showed that the mediating effect of the digital divide exists, and the confidence intervals do not contain 0.

**Table 3:** Results of bootstrap test

Path	Effect coefficient	Standard error	95% confidence interval	
			lower limit	upper limit
Direct effect				
Cultural capital - health of older adults	0.923^***^	0.094	0.740	1.106
Individual cultural capital - health of older adults	0.582^***^	0.085	0.416	0.748
Institutional cultural capital - health of older adults	0.490^***^	0.062	0.369	0.611
Cultural capital - health of older adults in urban areas	1.077^***^	0.126	0.830	1.324
Individual cultural capital - health of older adults in urban areas	0.527^***^	0.134	0.264	0.791
Institutional cultural capital - health of older adults in urban areas	0.694^***^	0.089	0.519	0.868
Cultural capital - health of older adults in rural areas	0.776^***^	0.140	0.502	1.049
Individual cultural capital - health of older adults in rural areas	0.933^***^	0.193	0.554	1.311
Institutional cultural capital - health of older adults in rural areas	0.342^***^	0.087	0.172	0.512
Indirect effect				
Cultural capital - digital divide - health of older adults	0.230	0.028	0.175	0.288
Individual cultural capital - digital divide - health of older adults	0.184	0.022	0.143	0.229
Institutional cultural capital - digital divide - health of older adults	0.139	0.016	0.109	0.170
Cultural capital - digital divide - health of older adults in urban areas	0.348	0.046	0.259	0.441
Individual cultural capital - digital divide - health of older adults in urban areas	0.441	0.051	0.340	0.541
Institutional cultural capital - digital divide - health of older adults in urban areas	0.222	0.027	0.171	0.277
Cultural capital - digital divide - health of older adults in rural areas	0.089	0.030	0.032	0.151
Individual cultural capital - digital divide - health of older adults in rural areas	0.126	0.041	0.050	0.210
Institutional cultural capital - digital divide - health of older adults in rural areas	0.057	0.017	0.025	0.093

*Note*: ^***^, ^**^, and ^*^ indicate 1%, 5%, and 10% significance levels, respectively; Bootstrap intervals are estimated as repeated self-sampling 1000 times 95% confidence intervals

### Moderating effects of cognitive ability

The results of the moderating effect test of cognitive ability are shown in Eq. 5 and Eq. 8 in Table 2. Equation 8 shows the moderating effect of cognitive ability between cultural capital and digital divide and it can be seen that cognitive ability positively moderates the relationship between cultural capital and digital divide (*β* = 0.072, *P* < 0.01). Equation 5 shows that the interaction term between digital divide and cognitive ability is not significant (*β* = 0.049, *P* > 0.1).

### Moderated mediation effect

Based on the previous Eqs. (2) and (3), the mediating effect of moderation can be confirmed by passing the significance test for any of the$$ {a}_{1}{b}_{2}$$, $$ {a}_{3}{b}_{1}$$, and $$ {a}_{3}{b}_{2}$$. $$ {a}_{1}$$ corresponds to the estimated coefficient of the digital divide for cultural capital in Eq. 8 of Table 2 ($$ {a}_{1}$$=0), *b*_2_ corresponds to the coefficient of the interaction term of the digital divide and cognitive ability in Eq. 5 of Table 2 ($$ {b}_{2}$$=0), the $$ {a}_{3}$$ corresponds to the coefficient of the interaction term between cultural capital and cognitive ability in Eq. 8 of Table 2 ($$ {a}_{3}$$≠0), and $$ {b}_{1}$$ corresponds to the estimated coefficient of the digital divide in Eq. 5 of Table 2 ($$ {b}_{1}$$≠0). Thus, it can be inferred that $$ {a}_{3}{b}_{1}$$≠0 and the mediation effect hypothesis with moderation holds. However, this method has the disadvantage of low testing power. In order to solve this problem, this paper uses SPSS PROCESS to calculate the value of the conditional indirect effect. The common practice is to select the mean plus or minus one standard deviation as the two ends of the value of the conditioning variable, respectively, to test the significance of the difference of the mediating effect under the conditioning value. The test results are shown in Table 4. When cognitive ability is at a lower level, the indirect effect of cultural capital on the health of older adults through the digital divide is significant, with an effect coefficient of 0.071 and a confidence interval of [0.050, 0.096], which does not contain 0. When cognitive ability is at a higher level, the indirect effect of cultural capital on the health of older adults through the digital divide is significant, with an effect coefficient of 0.264 and a confidence interval of [0.201, 0.333], not including 0. In addition, as shown in Table 5, the coefficient of determination INDEX is also significant, with an effect coefficient of 0.072 and a confidence interval of [0.052, 0.093], not including 0. This indicates that cognitive ability can modulate the mediating role of the digital divide in the relationship between cultural capital and the health of older adults, and the higher the level of cognitive ability, the more vital the mediating role of the digital divide.

**Table 4:** Mediation effect test results with moderation

Conditional indirect effect	Cognitive ability	Indirect effect value	Standard error	95% confidence interval	
				lower limit	upper limit
Cultural capital - digital divide - health of older adults	low value	0.071	0.012	0.050	0.096
	high value	0.264	0.034	0.201	0.333

*Note*: Bootstrap intervals are estimated as repeated self-sampling 1000 times 95% confidence intervals

**Table 5:** INDEX

Independent variables	Mediating variable	INDEX	Standard error	95% confidence interval	
				lower limit	upper limit
Cultural capital	Digital divide	0.072	0.010	0.052	0.093

*Note*: Bootstrap intervals are estimated as repeated self-sampling 1000 times 95% confidence intervals

### Endogeneity test

Although the estimation results of the above regression method can provide a general answer to the question of cultural capital affecting the health of older adults, due to the possible endogeneity problem caused by “sample bias,” in order to eliminate the endogeneity of the model as much as possible, this paper chooses to use the propensity score matching (PSM) to analyze with STATA [25]. In this paper, we take the cultural capital possessed by older adults as an example and estimate the average treatment effect on the treated (ATT) of cultural capital on the health of older adults by matching older adults in groups. The core of assessing the effect of matching is to compare the status of the control variables before and after matching. In this paper, cultural capital is a discrete variable that must be treated as a dichotomous variable to identify the control and treatment groups. According to the distribution of cultural capital scores in the sample, 0 is categorized as the “low cultural capital group” as the control group, and 1 and 2 are categorized as the “high cultural capital group” as the treatment group, thus constructing the treatment variables of PSM.

The balance test is mainly used to evaluate the matching effect by observing the standardized bias before and after matching [26]. Table 6 shows the results of the balance test of the data. The standardized bias of the two groups of control variables after matching is less than 5%, indicating that the matching effect can meet the requirements. The standardized bias of all the control variables has shrunk considerably, among which the marital abatement is the largest, reaching 99.2%; the income abatement is the smallest, only 76.8%. From the overall goodness-of-fit statistics of the model, it can be seen that the values of the control variables before and after matching are the same, indicating that the model fits well.

**Table 6:** Balance test results

Variables	Unmatched	Mean		%bias	%reduce	t-test	
	Matched	Treated	Control		| bias|	t	P >| t|
Household	U	0.570	0.377	39.5		15.50	0.000
	M	0.570	0.566	0.8	98.0	0.35	0.726
Gender	U	0.370	0.668	-62.3		-24.47	0.000
	M	0.370	0.367	0.8	98.7	0.35	0.724
Marriage	U	0.890	0.829	17.6		7.09	0.000
	M	0.890	0.889	0.1	99.2	0.07	0.941
Age	U	1.280	1.363	-15.3		-6.12	0.000
	M	1.280	1.288	-1.5	89.9	-0.74	0.460
Income	U	2.838	2.395	37.8		14.74	0.000
	M	2.838	2.735	8.8	76.8	4.09	0.000
Health insurance	U	0.918	0.883	11.7		4.69	0.000
	M	0.918	0.916	0.5	95.9	0.23	0.815
Smoke	U	0.322	0.214	24.6		9.55	0.000
	M	0.322	0.328	-1.3	94.7	-0.55	0.579
Drink	U	0.183	0.123	16.8		6.49	0.000
	M	0.183	0.184	-0.2	98.7	-0.09	0.926

*Note*: This table shows the test results for kernel matching; all other matching methods pass the balance test

Referring to the practice of related scholars [27], in this paper, the three methods of K-nearest neighbor matching, radius matching, and kernel matching are chosen to calculate the ATT of cultural capital on the health of older adults in turn, and the results are shown in Table 7. All three matching methods show that after matching, cultural capital has a significant positive effect on the health of older adults, and the ATT of the three matching methods are 0.704, 0.957, and 0.770, respectively. In summary, after the PSM test, the results of the promotional effect of cultural capital on the health of older adults are robust, and the potential risk of endogeneity is small.

**Table 7:** ATT effects of cultural capital on the health of older adults

Variables	Matching method	ATT	Standard error	t
Cultural capital	K-nearest neighbor	0.704^***^	0.122	5.75
	Radius	0.957^***^	0.036	26.32
	Kernel	0.770^***^	0.071	10.89

*Note*: ^***^, ^**^, and ^*^ indicate 1%, 5%, and 10% significance levels, respectively

### Robustness test

In this paper, the robustness test of the replacement variable is selected. In this paper, the hierarchical regression test is conducted with memory health as a replacement variable for the health of older adults. The regression results are presented uniformly with the previous paper to facilitate the observation of control with the research results. The robustness test results are consistent with the above conclusions, as seen in Table 8. The regression results of this paper are pretty robust.

**Table 8:** Robustness test results

Variables	Dependent variable: memory health				
	Equation 8	Equation 9	Equation 10	Equation 11	Equation 12
Cultural capital		0.832^***^ (0.052)	0.623^***^ (0.093)	0.657^***^ (0.054)	0.658^***^ (0.054)
Cognitive ability			0.056^***^ (0.017)	0.044^***^ (0.012)	0.039^***^ (0.013)
Cultural capital × cognitive ability			0.062^**^ (0.032)		
Digital divide				0.569^***^ (0.077)	0.451^**^ (0.175)
Digital divide × cognitive ability					-0.036 (0.048)
Control variables	Yes	Yes	Yes	Yes	Yes
R^2^	0.049	0.084	0.092	0.099	0.099
Adjusted R2	0.048	0.083	0.091	0.098	0.098
F	42.272^***^	67.307^***^	61.142^***^	66.256^***^	60.778^***^

*Note*: ^***^, ^**^, and ^*^ indicate 1%, 5%, and 10% significance levels, respectively

### Heterogeneity analysis

Table 9 shows the results of the test of household heterogeneity, from which it can be seen that cultural capital has a significant positive effect on the physical health, mental health, and memory health of urban older adults (*β* = 0.234, *P* < 0.01), (*β* = 0.115, *P* < 0.01), (*β* = 1.076, *P* < 0.01). Cultural capital also had a significant positive effect on physical health, mental health, and memory health of rural older adults (*β* = 0.206, *P* < 0.01), (*β* = 0.097, *P* < 0.1), (*β* = 0.562, *P* < 0.01). Table 10 shows the results of the gender heterogeneity test, from which it can be seen that cultural capital has a significant positive effect on physical health, mental health, and memory health of older adults in men (*β* = 0.370, *P* < 0.01), (*β* = 0.145, *P* < 0.01), (*β* = 0.729, *P* < 0.01). Cultural capital has a significant positive effect on the overall health of older adults of women (*β* = 0.950, *P* < 0.01), while there is no effect on the physical health and mental health of older adults of women.

**Table 9:** Results of the test for household heterogeneity

Household	Variables	Physical health	Mental health	Memory health
City	Cultural capital	0.234^***^ (0.066)	0.115^***^ (0.044)	1.076^***^ (0.072)
	Control variables	Control	Control	Control
	R^2^	0.034	0.024	0.086
Rural	Cultural capital	0.206^***^ (0.079)	0.097^*^ (0.055)	0.562^***^ (0.076)
	Control variables	Control	Control	Control
	R^2^	0.019	0.012	0.044

*Note*: ^***^, ^**^, and ^*^ indicate 1%, 5%, and 10% significance levels, respectively

**Table 10:** Results of the gender heterogeneity test

Gender	Variables	Physical health	Mental health	Memory health
Man	Cultural capital	0.370^***^ (0.068)	0.145^***^ (0.045)	0.729^***^ (0.073)
	Control variables	Yes	Yes	Yes
	R^2^	0.026	0.024	0.059
Woman	Cultural capital	0.040 (0.076)	0.051 (0.053)	0.950^***^ (0.075)
	Control variables	Yes	Yes	Yes
	R^2^	0.007	0.009	0.087

Note: ^***^, ^**^, and ^*^ indicate 1%, 5%, and 10% significance levels, respectively

## Discussion

### Cultural capital has a positive effect on the health of older adults

It was found that cultural capital favors the health of older adults, and the higher the level of cultural capital, the better the health of older adults, which is similar to the results of Wang Hb’s study [26]. In this study, the impact of cultural capital on the health of older adults was demonstrated through both individual cultural capital and institutional cultural capital. First, the formation of individual cultural capital is a continuous process of materialization and solidification of the flow of the individual through practical activities to receive an excellent artistic inculcation into their internal traits. Some studies have shown that good artistic inculcation can better stimulate the individual’s bodily perception and improve the individual’s immune ability [28]. Therefore, older adults can listen to musicals, watch art programs, and find other ways to relieve stress, relax, and maintain good health of older adults. Individual cultural capital has transmissibility, which can realize the transformation of social capital and promote the circulation of information between different levels. The process of participating in cultural activities is also the process of broadening one’s social network, and obtaining health knowledge at different levels can effectively reduce the problem of “information asymmetry.” Second, institutional cultural capital is an institutionalized state and manifests itself through an objectified form, institutional cultural capital is mainly manifested through the possession of education in previous studies, generally speaking, the higher the level of education an individual possesses, the higher the level of health literacy as well as the health needs of the individual, and then the better the individual’s health status will be [29]. The behaviors and attitudes of older adults’ learning also determine the degree of their desire for health, which is also important for promoting the improvement of the health of older adults [30].

### The digital divide mediates the role of cultural capital in influencing the health of older adults

The study found that the digital divide played a mediating role in the impact of cultural capital on the health of older adults. The size of the digital divide is expressed in terms of Internet use; the better the ability to use the Internet, the smaller the problem of the digital divide faced by older adults and the more effective it is in bridging it. Cultural capital is a prerequisite for older adults to bridge the digital divide and enhance Internet use. On the one hand, the characteristics and habits individuals possess impact the digital divide. Some studies have shown that the frequency of Internet use impacts the employment rate of the rural population, and the less frequent Internet use, the higher the unemployment rate of the rural population and the corresponding reduction in employment opportunities [31]. On the other hand, some studies have shown that the level of education affects the speed of individuals’ integration into the Internet era, and the higher the level of education, the faster the speed of individuals’ integration into the Internet era [32]. Some scholars even believe educational inequality is the culprit for the emergence of the digital divide problem [33]. Older adults realize the continuous transformation of social networks through the continuous accumulation of cultural capital, and older adults need to use the Internet when maintaining social networks, which prompts older adults to actively learn the skills related to Internet use, which not only maintains older adults’ motivation to use the Internet but also bridges the problem of digital divide among older adults to a certain extent. Bridging the issue of the digital divide among older adults has a favorable effect on the improvement of their health. One study found that the digital divide can have a negative impact on the health of older adults [34]. Yoon et al. found that Internet use is conducive to improving the health of older adults and that the digital divide is the leading cause of inequality in the health of older adults [35]. Internet use increases the frequency of learning among older adults, which in turn improves the health of older adults. The digital divide may have a negative impact on the health of older adults, while Internet use can have a positive impact, and the two are related but distinct from each other.

### Cognitive ability positively moderates the effect of cultural capital on the digital divide

Unlike previous studies, this study used cognitive ability as a moderating rather than predictive variable for improving the health of older adults. Cognitive ability positively moderates the relationship between cultural capital and the digital divide. On the one hand, with better cognitive ability, the positive effect of cultural capital on the digital divide will be strengthened. Generally speaking, older adults have better cognitive ability, which indicates a richer accumulation of cultural capital, and cultural capital can be transformed into social networks, further enhancing older adults’ cognitive ability. In this context, the problem of digital divide among older adults is further bridged. On the other hand, cognitive ability affects older adults’ use of the Internet, and the higher the level of cultural capital, the deeper the understanding of the importance of the Internet, the more conducive to promoting the active use of the Internet by older adults [36]. Therefore, good cognitive ability can enhance the level of cultural capital of older adults, making them more willing to accept and use the Internet, thus bridging the digital divide among older adults. It is worth noting that cognitive ability fails to modulate the impact of the digital divide on the health of older adults, possibly because the internal logic of the digital divide affecting the health of older adults is that older adults learn to use and utilize the Internet, thereby bridging the digital divide gap. However, due to their knowledge limitations and habituation to traditional lifestyles, the Internet is not important to some older adults, and they also lack motivation to learn, so the regulatory mechanism of cognitive ability fails to work.

### Cognitive ability positively moderates the mediating role of the digital divide between cultural capital influencing the health of older adults

In addition, cognitive ability was found to positively mediate the mediating role of the digital divide between cultural capital and the health of older adults. The higher the level of cognitive ability, the stronger the mediating role of the digital divide, thus the logic of cultural capital, digital divide, cognitive ability and health of older adults was further rationalized. With the rise of APP applications such as online pharmacies and online doctors, people can treat their diseases by buying medicines online and searching for doctors online, which brings great convenience to their daily lives. However, due to the long-term existence of older adults with low digital literacy and other reasons, older adults are unable to enjoy the convenient life brought by the Internet; furthermore, the Internet is filled with a variety of false and fraudulent information, and older adults have a higher probability of being cheated, which relies on the improvement of cognitive ability to help older adults to more quickly recognize, master and use the health of adults brought by the Internet [37]. Cognitive ability can process, recognize and deal with the information obtained from the Internet so that the vital information to maintain the individual’s health level can be gathered, the valuable information can be retained and the useless information can be discarded, i.e., “taking the best and removing the dross”.

### Cultural capital has a greater effect on the health of older adults among men in urban areas

From the household heterogeneity results, the impact of cultural capital on the health of older adults in urban areas is much more significant than on the health of older adults in rural areas. On the one hand, it may be because, due to the faster pace of urban development, the faster pace of life, and more opportunities for exposure to new things, older adults, in order to maintain their sense of social participation, will be able to adapt to the needs of social development by attending the University of the Elderly, participating in cultural activities and so on. On the other hand, cultural infrastructure construction is richer in cities than in rural areas, with libraries, science and technology museums, and other infrastructures able to help older adults accumulate cultural capital. At the same time, the popularization of the Internet has enabled older adults in cities to enjoy the conveniences of life brought about by the Internet at an earlier age and to protect significantly the health of older adults in cities using online medical consultations and the purchase of medicines, and so on. The gender heterogeneity results show that cultural capital affects the physical health, mental health, and overall health of older adults to a greater extent in men. First, it may be influenced by traditional thinking because the survey respondents in this paper are older adults over 60 years old. At that time, “valuing men over women” was deeply rooted in China, and women had fewer chances to receive education. Second, men have more opportunities to participate in social activities, which means they have access to various information channels and more information, such as health-related information. Therefore, the influence of cultural capital on the level of health of older adults is more significant for men.

### Policy implications

The findings of this study have important policy implications. From the perspective of cultural capital. On the one hand, the government should continue to improve the education and training system for the elderly to help older adults continuously improve their knowledge base, broaden their horizons, and keep pace with social development; on the other hand, the government should also endeavor to build an excellent cultural platform for the elderly. According to the interests of local older adults, the government should encourage communities to convene older adults to set up performing arts troupes and perform between communities, which will be conducive to the cultural enrichment of older adults and their maintaining optimism and improving the health of older adults. From the perspective of digital divide. First, the government should increase investment in digital infrastructure and products, continuously expand Internet penetration, and enhance older adults’ ability to access and use digital. Second, the community should also play a publicity role to help older adults recognize the changes that the Internet has brought to their lives through a combination of online and offline methods. Finally, families play an essential role in helping older adults improve their ability to use the Internet, and the help of their children will make older adults more interested in learning. Thus creating a good government-community-family integrated digital-friendly environment [38]. From the perspective of older adults themselves, older adults are at odds with the requirements of today’s era due to the decline in their physical functions, lower digital literacy, etc. In order to adapt to the needs of social development, older adults should maintain an active learning mindset and continuously improve their cultural capital, thus continuously improving their health of older adults.

### Strengths and limitations

This study has several strengths. For example, the study used the most recently released data, the data are current, and the findings can be generalized to relevant populations. The study examined factors such as cultural capital, digital divide, and cognitive ability, and to some extent, the relationship between these factors and the health of older adults can be assessed. However, this study has some limitations. For example, the cross-sectional data used in this study could not accurately capture deeper causal relationships between variables as well as the temporal relationships between variables, and therefore it would be more appropriate to use longitudinal data in subsequent studies. In addition, cultural capital is generally categorized into Individual cultural capital, Objective cultural capital and Institutional cultural capital, this study only elaborates on Individual cultural capital and Institutional cultural capital and ignores the role of Objective cultural capital, which should be avoided in future studies.

## Conclusion

This paper systematically explores the relationship between cultural capital and the health of older adults based on the stress coping theory and the data from the 2020 China Family Panel Studies (CFPS). It is found that cultural capital has a significant positive effect on the health of older adults, and the higher the level of cultural capital, the better the health of older adults. The digital divide has a mediating role between cultural capital and health of older adults, i.e., cultural capital reduces the digital divide gap among older groups by improving the literacy level of older adults in knowledge and technology, prompting them to learn and use the Internet, and then improving the health of older adults. Cognitive ability positively moderates the effect of cultural capital on the digital divide, while moderating the mediating role of the digital divide between cultural capital and the health of older adults. The effect of cultural capital on the health of older adults living in urban areas and being male was more pronounced in terms of health enhancement. Further research, such as longitudinal studies, is necessary to determine the causal logic between the variables in order to inform the improvement of the health of older adults in developing countries.

## Data Availability

All Publicly available datasets were analysed in this study. This data can be found at: http://isss.pku.edu.cn/cfps/.
